# Epidemiology of Australian Influenza-Related Paediatric Intensive Care Unit Admissions, 1997-2013

**DOI:** 10.1371/journal.pone.0152305

**Published:** 2016-03-29

**Authors:** Marlena C. Kaczmarek, Robert S. Ware, Mark G. Coulthard, Julie McEniery, Stephen B. Lambert

**Affiliations:** 1 Child Health Research Centre, School of Medicine, The University of Queensland, Brisbane, Australia; 2 The University of Queensland, School of Public Health, Brisbane, Australia; 3 Queensland Children’s Medical Research Institute, Brisbane, Australia; 4 Paediatric Intensive Care Unit, Lady Cilento Children’s Hospital, Brisbane, Australia; 5 Academic Discipline of Paediatrics and Child Health, School of Medicine, The University of Queensland, Brisbane, Australia; 6 Communicable Diseases Unit, Queensland Health, Brisbane, Australia; The University of Tokyo, JAPAN

## Abstract

**Background:**

Influenza virus predictably causes an annual epidemic resulting in a considerable burden of illness in Australia. Children are disproportionately affected and can experience severe illness and complications, which occasionally result in death.

**Methods:**

We conducted a retrospective descriptive study using data collated in the Australian and New Zealand Paediatric Intensive Care (ANZPIC) Registry of influenza-related intensive care unit (ICU) admissions over a 17-year period (1997–2013, inclusive) in children <16 years old. National laboratory-confirmed influenza notifications were used for comparison.

**Results:**

Between 1997 and 2013, a total of 704 influenza-related ICU admissions were recorded, at a rate of 6.2 per 1,000 all-cause ICU admissions. Age at admission ranged from 0 days and 15.9 years (median = 2.1 years), with 135 (19.2%) aged <6 months. Pneumonia/pneumonitis and bronchiolitis were the most common primary diagnoses among influenza-related admissions (21.9% and 13.6%, respectively). More than half of total cases (59.2%) were previously healthy (no co-morbidities recorded), and in the remainder, chronic lung disease (16.7%) and asthma (12.5%) were the most common co-morbidities recorded. Pathogen co-detection occurred in 24.7% of cases, most commonly with respiratory syncytial virus or a staphylococcal species. Median length of all ICU admissions was 3.2 days (range 2.0 hours– 107.4 days) and 361 (51.3%) admissions required invasive respiratory support for a median duration of 4.3 days (range 0.2 hours– 107.5 days). There were 27 deaths recorded, 14 (51.9%) in children without a recorded co-morbidity.

**Conclusion:**

Influenza causes a substantial number of ICU admissions in Australian children each year with the majority occurring in previously healthy children.

## Introduction

Influenza virus causes a significant burden of disease annually in Australia [1]. Influenza activity varies from year to year and is dependent on the circulating viruses and prevailing population immunity. More severe seasons, such as during 2009, are often associated with novel viruses to which there is little or no prior immunity at the population level [2, 3].

Clinically, influenza infection spans a wide severity spectrum, from asymptomatic infection or mild illness, through to severe disease and death [2]. Severe illness occurs most commonly among children, the elderly, and individuals with pre-existing chronic medical conditions [2]. In infants and children, influenza can cause complications such as croup, bronchiolitis, exacerbation of asthma, pneumonia, and seizures, all of which are associated with hospitalisation and intensive care unit (ICU) admission [2, 4, 5]. In Australia, the age-specific burden of disease is U-shaped, with the highest rates of notifications and hospitalisations occurring in children aged under 5 years and in adults aged 65 years or greater [1].

Annual influenza immunisation is currently recommended for all individuals aged ≥6 months for whom it is desired to reduce the likelihood of becoming ill with influenza [6]. However Government funding for free vaccination under the Australian National Immunisation Program is currently limited to adults ≥65 years old, Aboriginal and Torres Strait Islander peoples aged ≥6 months to <5 years and ≥15 years, pregnant women, and individuals ≥6 months old at risk of severe illness due to medical conditions [6, 7].

Influenza activity is monitored primarily through reporting of laboratory confirmed cases under public health legislation [8], which does not routinely capture details around the severity of illness. Investigation of severe paediatric influenza-related illness, focussing only on hospitalisations and ICU admissions, has primarily been reported at the Australian state level or nationally only during peak years (such as the 2009 influenza pandemic) [9–13]. The aim of this study is to describe the epidemiology of severe paediatric influenza-related admissions to all major Australian paediatric ICUs over a 17 year period.

## Methods

We conducted a retrospective descriptive study using data collated in the Australian and New Zealand Paediatric Intensive Care (ANZPIC) Registry. The ANZPIC Registry, established in 1997, collects paediatric intensive care patient episode information from contributing specialist paediatric ICUs (PICUs) as well as general ICUs across Australia and New Zealand [14]. In 1997, eight PICUs from five Australian states (NSW, QLD, SA, VIC, WA) contributed data to the ANZPIC Registry [14]. By 2013, one additional PICU and 11 ICUs (representing all states/territories) were participating. The original eight PICUs, of which seven have contributed data continuously since commencement, accounted for an annual average of 93% of admissions recorded in the ANZPIC Registry between 1997 and 2013 (1997 value: 100%, 2013 value: 84%).

Participating ICUs collect data in real-time either on a Microsoft Access database (Microsoft Corp, Redmond, WA, USA) supplied by the ANZPIC Registry or onto their local clinical information system (amended to include required ANZPIC Registry fields). Data are submitted to the centrally administered ANZPIC Registry electronically every six months [14].

A single record is created for every admission to a participating ICU and includes demographic data, physiological variables measured at the time of first face-to-face contact between the patient-ICU doctor, the ICU/hospital outcomes and length of stay, as well as the type and duration of respiratory support [14]. Admissions are defined using ANZPIC Registry-specific standardised diagnosis codes [15] in up to 9 diagnostic fields including ‘principal diagnosis’ (the diagnosis most directly responsible for the ICU admission), ‘underlying diagnosis’ (the principal underlying diagnosis contributing to the need for ICU admission), and up to 7 ‘associated diagnoses’. Associated diagnoses are conditions additional to the principal and underlying reasons that contributed to the ICU admission, and can include other syndromes, diseases, abnormalities or diagnoses identified on or during ICU admission. ANZPIC Registry coding requires infection diagnosis codes (700–799) to be used only in the ‘underlying’ or ‘associated’ diagnosis fields and not in the ‘principal diagnosis’ field. The treating clinician is responsible for selecting the appropriate diagnostic codes for each admission, and coding may be based on clinical symptoms, diagnostic test results, or a combination of these. Results of any diagnostic tests performed, including respiratory virus/bacteria detection or influenza type/subtype analysis, are not captured within the ANZPIC Registry. Immunisation history is also not collected.

For this study, we extracted ANZPIC Registry data for all Australian paediatric (aged 0 to <16 years) ICU admissions (New Zealand admissions not included) between 01 January 1997 and 31 December 2013 with a diagnosis code of “715 –Influenza Virus” occurring in any of the diagnostic fields. The de-identified line-listed data extract included patient demographic variables (age, sex, ethnicity, state), and hospital and ICU admission details—admission/discharge dates, diagnoses (principal, underlying, and associated), discharge/outcome, and respiratory support variables (nature and duration of respiratory support). Respiratory support was defined as any intervention to support respiratory function and includes both non-invasive and invasive methods. Some patients may have required combinations of both invasive and non-invasive support during their ICU admission. Non-invasive respiratory support includes continuous positive airway pressure, biphasic positive airway pressure, negative pressure ventilation, and high flow nasal cannula, while invasive respiratory support is mechanical ventilation delivered by endotracheal intubation or tracheostomy.

Data were aggregated during analysis, including grouping by year and age. Admissions were classified has having co-morbidities if they had a diagnosis code (in any diagnostic field) for any co-morbid condition defined in the Australian Immunisation Handbook as predisposing individuals to severe influenza, and therefore allowing publicly-funded vaccine use, including cardiac disease or congenital cardiovascular conditions, asthma, chronic respiratory disease, cystic fibrosis, immunodeficiency or immunosuppression, Down syndrome, chronic neurological conditions, diabetes, chronic renal failure, haemoglobinopathies, and chronic inherited metabolic disorders [6]. Admissions were classified as having co-detection if they had any other respiratory infection diagnosis code in any diagnostic field (in addition to an influenza code).

In Australia, diagnostic testing for influenza is funded under the Medical Benefits Scheme when required for medical management of a patient. As laboratory confirmed influenza has been a nationally notifiable condition since 2001, where testing is performed, all cases that meet the case definition are required to be reported to the appropriate State or Territory Health Department in accordance with public health legislation [8]. The National Notifiable Diseases Surveillance System (NNDSS) compiles information collected by State and Territory Health Departments into one passive national system. National influenza notification data for children <16 years old were obtained from the NNDSS for this study. Using mid-year Australian Bureau of Statistics population estimate data, we calculated the influenza notification rate in children <16 years per 100,000 population, and compared this with national influenza-related paediatric ICU admissions per 1,000 all-cause paediatric ICU admissions, as well as per 100,000 population.

Stata version 12 (StataCorp, College Station, TX, USA) was used for data analysis. Descriptive statistics were calculated, including means, medians, and standard deviations (SD). The association between patient characteristics and outcomes was investigated using Student’s t-test. The correlation between influenza-related ICU admissions and influenza notifications was calculated in Microsoft Excel (Microsoft, Redmond, WA, USA).

The Children’s Health Services Queensland Human Research Ethics Committee granted ethics approval for this study, including a waiver of individual consent due to the data being de-identified.

## Results

**Fig 1:**
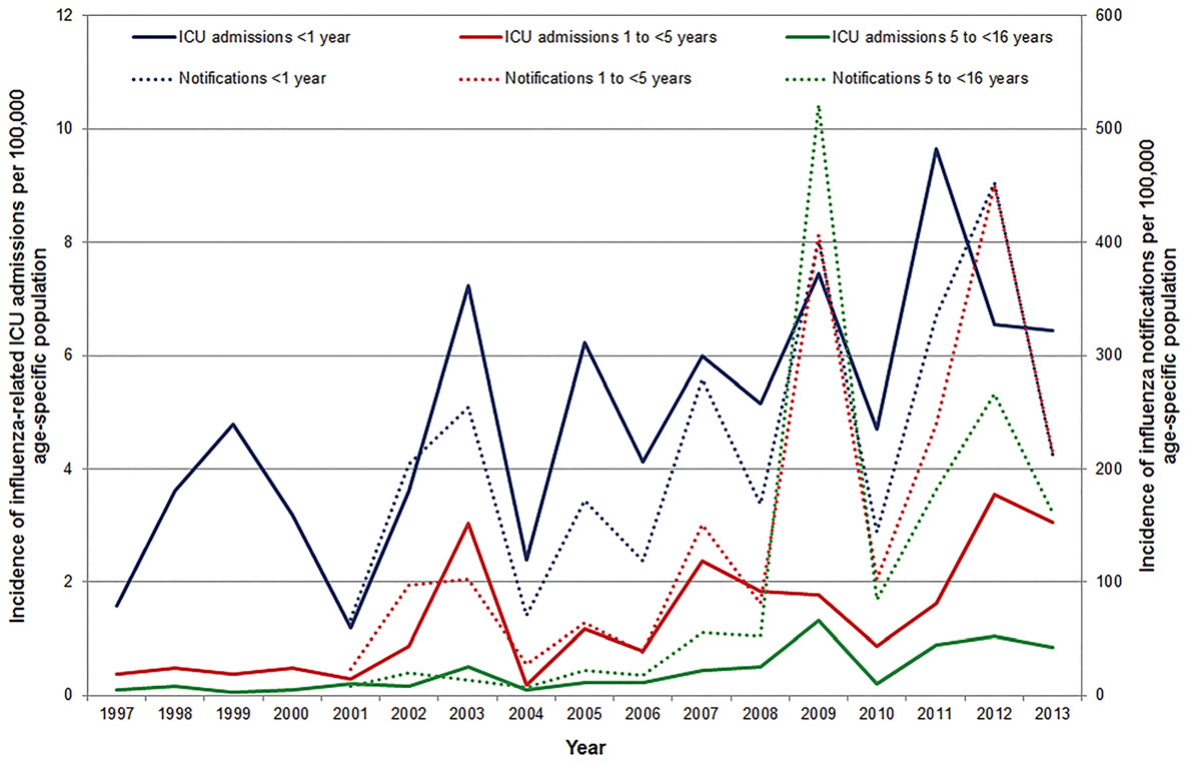
Incidence of influenza-related ICU admissions (primary axis) and influenza notifications* (secondary axis), per 100,000 age-specific population, Australia, 1997–2013*, by age group and year. * *Influenza became nationally notifiable in 2001 –notification data were not nationally collected prior to 1 January 2001*.

Between 01 January 1997 and 31 December 2013, 704 influenza-related ICU admissions were identified in the ANZPIC Registry of a total 113,197 paediatric ICU admissions from the same ICUs during the period (Table 1). This gives an average proportion for the entire study period of 6.2 influenza-related ICU admissions per 1,000 all-cause ICU admissions (95% CI: 5.8 to 6.7), however this proportion varied between years, peaking in 2003, 2009, and 2012 at 10.3, 11.4, and 11.3 per 1,000 ICU admissions, respectively (Fig 1). Annual influenza notifications also peaked in 2009 and 2012 at 484.2 and 326.2 per 100,000 population, respectively. Both influenza ICU admissions and notifications in children <16 years generally increased over the study period and the two datasets were correlated (correlation coefficient = 0.68).

**Table 1:** Demographics and admission characteristics of influenza-related ICU admissions, 1997–2013.

	Total *
**Number of participating ICUs** ^†^	Between 7 and 21
**Influenza-related admissions**	704
*Influenza-related admissions per 1000 all-cause ICU admissions (total ICU admissions)*	6.2 (113,197)
**Sex**	
*MALE—Number (percent)*	400 (56.8%)
**Ethnicity**	
*Aboriginal and Torres Strait Islander peoples—Number of recorded (percent)*	19 of 282 (6.7%)
**Age**	
Aged <6 months	135 (19.2%)
Aged 6 months to <5 years	353 (50.1%)
Aged 5 years to <16 years	216 (30.7%)
*Median (range)*	2.1 years
	(0 days—15.9 years)
**Location (state/territory)** ^‡^	
*Number (percent of total influenza admissions); total all-cause ICU admissions*	
Australian Capital Territory	9 (1.3%); 369
New South Wales	273 (39.1%); 32,950
Northern Territory	11 (1.6%); 548
Queensland	171 (24.5%); 28,902
South Australia	38 (5.4%); 9,514
Tasmania	14 (2.0%); 1,631
Victoria	137 (19.6%); 26,337
Western Australia	45 (6.5%); 12,946
**Patients with co-morbidities**	
*Number (percent)*	287 (40.8%)
**Patients with infectious agent co-detection**	
*Number (percent)*	174 (24.7%)
**Length of hospitalisation**^§^	9.6 days
*Median (range)*	(6.2 hours—248.6 days)
**Length of ICU stay**	3.2 days
*Median (range)*	(2.0 hours—107.5 days)
**Any respiratory support during 1**^**st**^ hour of ICU admission** ^††^	
*Number (percent)*	355 (52.2%)
Any respiratory support during ICU admission** ^††^	
*Number (percent)*	418 (66.8%)
**Invasive respiratory support during ICU admission**^‡‡^	
*Number (percent)*	361 (51.3%)
**Length of invasive respiratory support**	4.3 days
*Median (range)*	(0.2 hours—107.5 days)
**Deaths**	
*Number with co-morbidities (percent of cases with co-morbidities)*	13 (4.5%)
*Number without co-morbidities (percent of cases without co-morbidities)*	14 (3.4%)

* For analysis by year see Supporting Information (S1 Table. Demographics and admission characteristics of influenza-related ICU admissions, 1997–2013)

^†^ The number of participating ICUs varied over time.

^‡^ Three ICU admissions had no residential state recorded, and three were classified as residing overseas

^§^ Hospitalisation duration data missing for 98 cases

** Respiratory support is defined as any intervention to support respiratory function and includes both non-invasive and invasive methods. Some patients may have required combinations of both invasive and non-invasive support during their ICU admission. Non-invasive respiratory support includes: continuous positive airway pressure, biphasic positive airway pressure, negative pressure ventilation, and high flow nasal cannula.

^††^ Respiratory support in 1^st^ hour data missing for 24 cases; Respiratory support during admission data missing for 78 cases.

^‡‡^ Invasive respiratory support is mechanical ventilation delivered by endotracheal intubation or tracheostomy.

**Fig 2:**
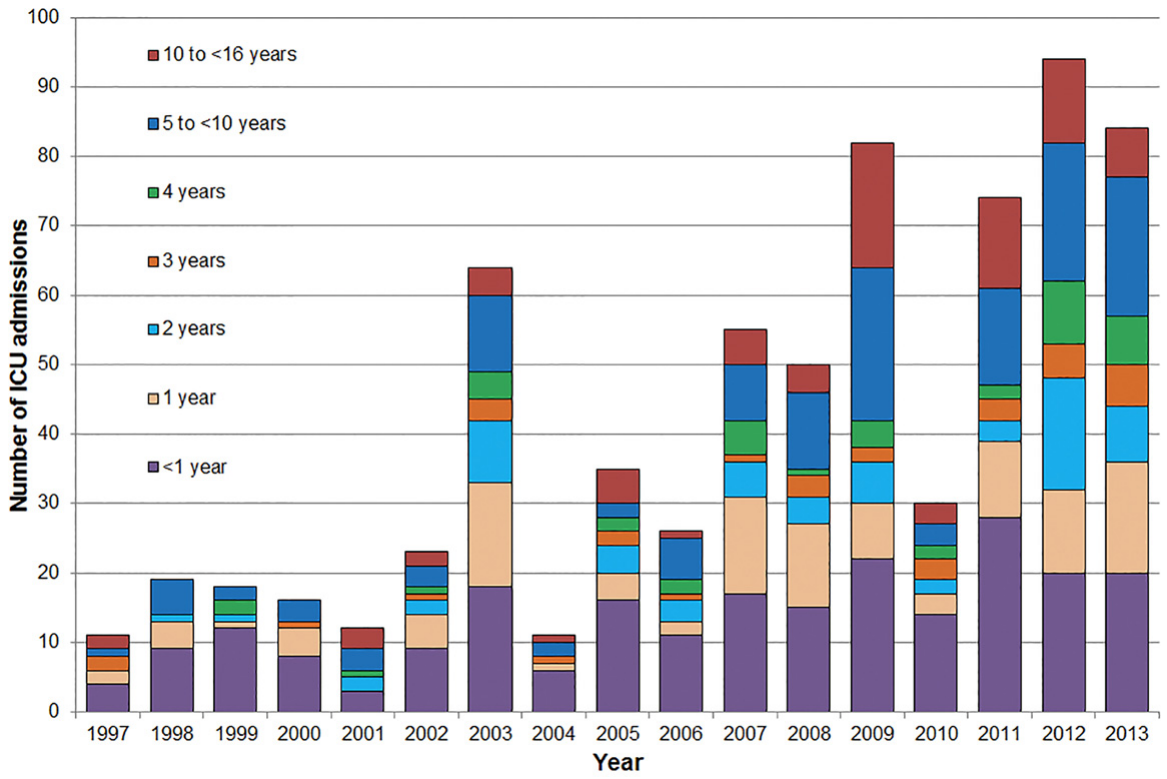
Paediatric (0 to <16 years) influenza-related ICU admissions, Australia, 1997–2013, by year and age at admission.

Over the study period, a higher proportion of ICU admissions were male (56.8%), and 6.7% of cases where ethnicity data were available (n = 282) were identified as Aboriginal or Torres Strait Islander people (Table 1). The median age of children admitted with influenza was 2.1 years (range: 0 days to 15.9 years), however there was some variation over the study period (S1 Table). Compared with the 1997–2008 period, the mean age of ICU admissions was significantly higher after 01 January 2009, (mean (SD) age: 3.3 (3.8) years vs 4.5 (4.4) years, mean difference (MD) = 1.2 years, 95%CI: 0.6 to 1.8, p = 0.0001). Across all years, admission counts were highest for children in their first year of life (Fig 2). Overall, children <1 year old accounted for 11.1% of influenza notifications (aged <16 years) and 33.0% of paediatric influenza-related ICU admissions.

Of the 704 admissions, two (0.3%) had influenza coded in the principal diagnosis field, 87 (12.4%) in the underlying diagnosis field, and 617 (87.6%) in one of the associated diagnosis fields (two cases had the code included in both the underlying and an associated diagnosis field). The most common principal diagnoses for influenza ICU admissions were: pneumonia/pneumonitis (n = 154, 21.9%), bronchiolitis (n = 96, 13.6%), seizures (n = 70, 9.9%), croup (n = 43, 6.1%), and respiratory failure (n = 40, 5.7%). Of 232 cases aged <1 year, 50 (21.6%) had prematurity as an underlying or associated diagnosis code, of which 26 (52.0%) were aged <6 months and 24 (48.0%) were aged between 6 months and <1 year.

Immunisation Handbook defined co-morbidities [6] were coded in 40.8% (n = 287) of cases, most commonly chronic lung disease (includes bronchopulmonary dysplasia) (n = 48, 16.7%) and asthma (n = 36, 12.5%). Relatively few of the 135 infants aged ≤6 months had co-morbidities (30.4%), with the proportion of admissions with co-morbidities increasing with age (37.1% of 6 month to <5 year olds; 53.2% of 5 to <16 year olds). Compared to previously healthy children, cases with co-morbidities were significantly older, stayed in ICU longer, and, when needed, required respiratory support for a longer period (Table 2).

**Table 2:** Comparisons between groups with/without co-morbidities or with/without co-detection, influenza-related ICU admissions, 1997–2013.

	Comparison of interest	WITH	WITHOUT	Mean difference	Odds Ratio	95% CI	P value
		*Mean (SD)*	*Mean (SD)*				
**Co-morbidities**	Age	4.7 (4.3) years	3.3 (4.0) years	1.4 years	-	0.8 to 2.0 years	<0.0001
	Length of ICU stay	7.1 (9.7) days	5.0 (7.5) days	2.1 days	-	0.8 to 3.3 days	0.002
	Need for respiratory support	-	-	-	1.1	0.8 to 1.6	0.55
	Duration of any respiratory support (mean length)*	5.2 (9.2) days	3.4 (7.4) days	1.9 days	-	0.5 to 3.2 days	0.006
	Duration of invasive respiratory support (mean length)^†^	3.4 (6.3) days	3.1 (7.1) days	0.4 days	-	-0.6 to 1.4 days	0.45
	Death	-	-	-	1.4	0.6 to 3.2	0.43
**Co-detection**	Age	3.7 (4.3) years	4.0 (4.1) years	0.3 years	-	0.4 to 1.0 years	0.46
	Length of ICU stay	8.5 (9.0) days	5.0 (8.1) days	3.5 days	-	2.1 to 5.0 days	<0.0001
	Need for respiratory support	-	-	-	1.8	1.2 to 2.8	0.005
	Duration of any respiratory support (mean length)*	8.1 (8.0) days	5.4 (9.8) days	2.6 days	-	0.7 to 4.6 days	0.009
	Duration of invasive respiratory support (mean length)^†^	8.2 (7.5) days	5.5 (8.7) days	2.8 days	-	0.9 to 4.7 days	0.005
	Death	-	-	-	0.5	0.2 to 1.5	0.26

* Respiratory support is defined as any intervention to support respiratory function and includes both non-invasive and invasive methods. Some patients may have required combinations of both invasive and non-invasive support during their ICU admission. Non-invasive respiratory support includes: continuous positive airway pressure, biphasic positive airway pressure, negative pressure ventilation, and high flow nasal cannula.

^†^ Invasive respiratory support is mechanical ventilation delivered by endotracheal intubation or tracheostomy.

Co-detection (having a second infection diagnosis code), was common, with 24.7% (n = 174) of influenza-related ICU admissions. Respiratory syncytial virus (RSV) was most frequent among co-infected influenza cases (n = 46, 26.5%). Bacterial co-detection was recorded among 12.8% of cases (n = 22), with 26.7% (n = 6) of these coded as a staphylococcal species. There was no difference in the age of co-infected and influenza-only cases (p = 0.46), however co-infected cases had a significantly longer length of ICU stay compared to non-co-infected cases (MD = 3.5 days, 95%CI: 2.1 to 5.0 days, p<0.0001, Table 2). Co-detection was also associated with significant differences in the need for and mean length of respiratory support, both any and invasive, compared to cases only diagnosed with influenza (p = 0.009 and p = 0.005, respectively).

Overall, influenza cases stayed in ICU for a median of 3.2 days (range: 2.0 hours to 107.5 days) and in hospital for a median of 9.6 days (range: 6.2 hours to 248.6 days) (Table 1). There was no significant variation in the length of ICU or hospital stay over time (ICU p = 0.26, hospital p = 0.09) or by age (ICU p = 0.39, hospital p = 0.26) (see S1 Table). Most cases (78.5%) spent ≤7 days in ICU and another 13.2% were discharged from ICU in the subsequent 7 days.

Overall, 66.8% of cases required some form of respiratory support during their ICU admission, for a median length of 3.5 days (range: 0.2 hours to 107.5 days), with 52.2% receiving support during the first hour of ICU admission (Table 1). Approximately half of the admissions (51.3%) required invasive respiratory support, for a median length of 4.3 days (range: 0.2 hours to 107.5 days). There was no significant variation in the length of any respiratory support or length of invasive respiratory support by year (p = 0.76 and p = 0.60, respectively) or by age (p = 0.94 and p = 0.67, respectively).

A total of 27 deaths were recorded over the study period, which equated to 3.8% of all influenza-related ICU admissions (Table 1). The median age of children who died was 4.7 years (range: 48 days to 14.5 years). The highest number of deaths occurred in 2003 (n = 7, 10.9%). Among cases without co-morbidities (n = 417) there were 14 deaths (3.4%), compared with 13 deaths among cases with co-morbidities (n = 287, 4.5%). Chronic encephalopathy was the most common co-morbidity coded for deceased cases (n = 7), followed by chronic lung disease (n = 3). Among deceased cases, four children (14.8%) had co-detection of a second respiratory infection, of which two were RSV and two were *Pseudomonas*.

## Discussion

Over half the 704 influenza-related ICU admissions recorded in the ANZPIC Registry between 1997 and 2013 were previously healthy children with no co-morbidity. All children with influenza-related ICU admissions required long hospitalisations and ICU stays, and many required invasive respiratory support for considerable lengths of time, regardless of whether they had co-morbid conditions. Infants aged <1 year were more frequently represented among influenza-related ICU admissions than among influenza notifications, suggesting that younger infants are more vulnerable to severe disease and is consistent with findings from previous studies [9, 11]. We also identified that infants who had been born prematurely were over-represented among influenza-related ICU admissions (1 in 5) compared to the Australian population incidence of prematurity (approximately 1 in 10 live births) [16].

Severe paediatric influenza-related illness occurs in Australia, even in years with low influenza activity. In this study we have only focused on ICU admissions, however it is estimated that between 10–30% of influenza-related hospitalisations result in an ICU admission, depending on the season [12, 17–22]. Consistent with other reports of Australian paediatric influenza-related ICU admissions [9–13], we found a median length of stay of approximately 3 days and that half of the admissions required invasive respiratory support. The number of ICU admissions, as well as the high level of support required, highlights the substantial burden of influenza-related illness in children on the Australian healthcare system.

Findings from our study are a likely underestimate of disease burden. During the early years of our study period, only influenza-related cases admitted to participating ICUs were included, therefore any admissions at non-participating ICUs would have been missed. By 2013, the ANZPIC Registry captured 92.4% of all paediatric ICU admissions in Australia and New Zealand [14], and ANZPIC Registry data had become increasingly representative of total Australian ICU admissions as the number of participating ICUs increased over the study period. Additionally, as our study used data from an existing database and did not include a review of medical charts or diagnostic testing results, we were reliant on the accuracy of existing coding. Any admissions due to, but not coded as ‘influenza’, and those not coded with existing co-detections and co-morbidities, will have been misclassified. Identifying influenza and recording of the related diagnostic code is dependent on clinician awareness and testing, as well as the perceived importance of the illness/diagnosis, which could all vary between seasons [23, 24]. Improvements in diagnostic testing, with the widespread use of PCR from 2007 onwards [25], and high media coverage in some seasons (e.g. 2007 due to several influenza-related child deaths [26], influenza pandemic in 2009) may have influenced influenza testing and increased awareness among clinicians, primarily during the latter part of our study period. We cannot know whether the demographic profile and severity of missing cases was different from those included in our analysis, and the number of missing admissions, if any, cannot be quantified. Furthermore, due to the unavailability of typing results within the ANZPIC Registry, we were unable to investigate seasonal trends by influenza type.

As previously healthy children and those with co-morbidities were equally represented among influenza-related ICU admissions and deaths, more emphasis should be placed on annual influenza vaccination for all children. This has already been recognised in the United States and United Kingdom, with influenza vaccination universally recommended for all children [27, 28]. It has been estimated that fully vaccinated children are between 74–86% less likely to have an influenza-related hospital or ICU admission compared to unvaccinated children [10, 29, 30] and immunisation has been shown to reduce the burden of influenza in the community [13, 31, 32]. However influenza vaccination coverage among children in Australia remains low, even among individuals eligible for government-funded vaccination [6, 11, 12, 29]. Vaccination history is not collected in the ANZPIC Registry, however given the low influenza vaccine coverage among Australian children [29], it is unlikely that many (if any) of our ICU cohort were vaccinated. Australian primary care providers and paediatricians could improve coverage by discussing influenza vaccination with the parents of all their paediatric patients, including those that are otherwise healthy. Such discussions would be assisted by firming up the language used in the Australian Immunisation Handbook to be more aligned to that used in the United States: “Routine annual influenza vaccination is recommended for all persons aged ≥6 months who do not have contraindications”[27] and by expanding funding to provide free vaccine to all children aged <5 years, if not all children aged <17 years like in the United Kingdom [28].

Additionally, as approximately 20% of all influenza-related ICU admissions (including 50% of those coded as preterm infants) occurred in infants too young to be vaccinated (<6 months old), maternal vaccination should also be emphasised. Influenza vaccination during pregnancy has been shown to be safe and in addition to protecting the mother from severe infection, it also appears to reduce the likelihood of premature birth and subsequent influenza infection in the infant [33, 34]. Maternal vaccination has been recommended in Australia since March 2000, with funded vaccine available through the National Immunisation Program from January 2010 [35]. Although national coverage estimates are not available [36], state-based cross-sectional surveys have estimated that approximately 25–30% of women receive the vaccine during pregnancy [34, 37, 38]. Education, provided by a health care professional, about the possible severity of influenza infection, as well as the efficacy and safety of influenza vaccination, could improve uptake among children and pregnant women [33, 34, 37, 38]. As coverage in Australia increases, the impact on paediatric influenza-related hospitalisations and ICU admissions should be monitored.

## Conclusion

In Australia, influenza causes a substantial number of ICU admissions just over half of which occur in previously healthy children without documented co-morbidities. Lack of co-morbidities was also identified in half of all deaths. In the clinical setting, consideration should be given to recommending influenza vaccination to all children and pregnant women to prevent the severe outcomes associated with influenza infection, including hospitalisations and ICU admissions, among children.

## Supporting Information

S1 Table Demographics and admission characteristics of influenza-related ICU admissions, 1997–2013. (PDF)
