# Supplementary material for: Epidemiology of Australian Influenza-Related Paediatric Intensive Care Unit Admissions, 1997-2013
Source: PLoS One. 2016 Mar 29;11(3):e0152305. doi: 10.1371/journal.pone.0152305 (PMC4811543; doi:10.1371/journal.pone.0152305)
Supplement: S1 Table — (PDF) [file pone.0152305.s001.pdf]

Supplementary Table: Demographics and admission characteristics of influenza-related ICU admissions, 1997-2013

**Year of admission**	1997	1998	1999	2000	2001	2002	2003	2004	2005	2006	2007	2008	2009	2010	2011	2012	2013	Total
**Number of participating ICUs**	8	7	9	9	9	13	14	14	13	15	18	19	20	19	19	20	21	N/A
**Influenza admissions**<br>*Number (percent of total influenza admissions)*	11 (1.6)	19 (2.7)	18 (2.6)	16 (2.3)	12 (1.7)	23 (3.3)	64 (9.1)	11 (1.6)	35 (5.0)	26 (3.7)	55 (7.8)	50 (7.1)	82 (11.7)	30 (4.3)	74 (10.5)	94 (13.4)	84 (11.9)	704 (100)
Influenza per 1000 all-cause ICU admissions<br>*(total ICU admissions)*	2.1<br>(5,316)	4.0<br>(4,728)	3.2<br>(5,639)	2.9<br>(5,580)	2.0<br>(5,888)	3.8<br>(5,993)	10.3<br>(6,204)	1.8<br>(6,278)	5.6<br>(6,226)	3.8<br>(6,824)	7.8<br>(7,079)	6.9<br>(7,222)	11.4<br>(7,222)	3.9<br>(7,598)	9.5<br>(7,805)	11.3<br>(8,329)	9.1<br>(9,232)	6.2<br>(113,197)
**Sex**<br>*MALE - Number (percent)*	8 (72.7)	10 (52.6)	13 (72.2)	7 (43.8)	5 (41.7)	12 (52.2)	38 (59.4)	7 (63.6)	20 (57.1)	16 (61.5)	31 (56.4)	22 (44.0)	35 (42.7)	18 (60.0)	51 (68.9)	56 (59.6)	51 (60.7)	400 (56.8)
**Ethnicity**<br>*Aboriginal and Torres Strait Islander peoples – Number of recorded (percent)*	1 of 11<br>(9.1)	1 of 19<br>(5.3)	1 of 17<br>(5.9)	3 of 16<br>(18.8)	1 of 12<br>(8.3)	0 of 23<br>(0)	5 of 64<br>(7.8)	0 of 11<br>(0)	3 of 35<br>(8.6)	0 of 13<br>(0)	0 of 0<br>(0)	0 of 0<br>(0)	0 of 0<br>(0)	1 of 3<br>(33.3)	2 of 12<br>(16.7)	0 of 24<br>(0)	1 of 22<br>(4.6)	19 of 282<br>(6.7)
**Age**																		
Aged <6 months	4 (36.4)	7 (36.8)	11 (61.1)	6 (37.5)	1 (8.3)	7 (30.4)	9 (14.1)	2 (18.2)	7 (20.0)	5 (19.2)	10 (18.2)	7 (14.0)	13 (15.9)	6 (20.0)	16 (21.6)	12 (12.8)	12 (14.3)	135 (19.2)
Aged 6 months to <5 years	4 (36.4)	7 (36.8)	5 (27.8)	7 (43.8)	5 (41.7)	11 (47.8)	40 (62.5)	6 (54.6)	21 (60.0)	14 (53.9)	32 (58.2)	28 (56.0)	29 (35.4)	18 (60.0)	31 (41.9)	50 (53.2)	45 (53.6)	353 (50.1)
Aged 5 years to <16 years	3 (27.3)	5 (26.3)	2 (11.1)	3 (18.7)	6 (50.0)	5 (21.7)	15 (23.4)	3 (27.3)	7 (20.0)	7 (26.9)	13 (23.6)	15 (30.0)	40 (48.8)	6 (20.0)	27 (36.5)	32 (34.0)	27 (32.1)	216 (30.7)
*Median (range)**	1.4 years<br>(31d -<br>13.1y)	1.1 years<br>(31d -<br>9.0y	0.4 years<br>(16d -<br>10.0y)	1.1 years<br>(1d -<br>8.3y)	5.3 years<br>(106d -<br>14.6y)	1.4 years<br>(70d -<br>14.4y)	1.9 years<br>(0d -<br>13.9y)	1.0 years<br>(30d -<br>13.3y)	1.3 years<br>(27d -<br>13.2y)	1.7 years<br>(29d -<br>13.7y)	1.5 years<br>(32d -<br>13.8y)	1.9 years<br>(50d -<br>15.9y)	4.6 years<br>(16d -<br>15.7y)	1.2 years<br>(42d -<br>14.5y)	1.8 years<br>(0d -<br>14.8y)	2.9 years<br>(33d -<br>15.9y)	2.8 years<br>(11d -<br>14.8y)	2.1 years<br>(0d -<br>15.9y)
**Patients with co-morbidities**<br>*Number (percent)*	7 (63.6)	5 (26.3)	3 (16.7)	6 (37.5)	7 (58.3)	4 (17.4)	31 (48.4)	4 (36.4)	14 (40.0)	13 (50.0)	14 (25.5)	18 (36.0)	38 (46.3)	11 (36.7)	40 (54.1)	42 (44.7)	30 (35.7)	387 (40.8)
**Patients with co-detection**<br>*Number (percent)*	3 (27.3)	6 (31.6)	0 (0)	2 (12.5)	1 (8.3)	7 (30.4)	11 (17.2)	3 (27.3)	8 (22.9)	5 (19.2)	19 (34.6)	17 (34.0)	14 (17.1)	6 (20.0)	28 (37.8)	21 (22.3)	23 (27.4)	174 (24.7)
**Length of hospitalisation†**<br>*Median (range)*	13.8 days<br>(1.8d-<br>36.8d)	4.6 days<br>(2.2d-<br>13.1d)	7.7 days<br>(20.4h-<br>28.4d)	13.5 days<br>(5.4d-<br>28.9d)	5.5 days<br>(4.3d-<br>11.0d)	15.1 days<br>(1.4d-<br>62.0d)	6.6 days<br>(1.4d-<br>123.3d)	4.0 days<br>(2.5d-<br>8.0d)	14.8 days<br>(17.0h-<br>43.0d)	17.1 days<br>(1.0d-<br>31.3d)	7.8 days<br>(16.3h-<br>57.1d)	12.6 days<br>(2.0d-<br>119.0d)	10.8 days<br>(2.8d-<br>52.8d)	9.2 days<br>(1.2d-<br>46.6d)	9.1 days<br>(1.0d-<br>149.1d)	8.7 days<br>(6.2h-<br>248.6d)	9.1 days<br>(1.0d-<br>123.3d)	9.6 days<br>(6.2h-<br>248.6d)
**Length of ICU stay**<br>*Median (range)*	1.8 days<br>(6.0h-<br>14.5d)	2.5 days<br>(3.0h-<br>13.4d)	1.8 days<br>(7.0h-<br>19.8d)	4.2 days<br>(1.3d-<br>16.5d)	2.3 days<br>(5.0h-<br>14.8d)	3.8 days<br>(13.0h-<br>28.9d)	2.5 days<br>(6.0h-<br>20.5d)	2.7 days<br>(5.0h-<br>26.6d)	4.1 days<br>(5.0h-<br>43.1d)	2.7 days<br>(13.0h-<br>18.9d)	2.7 days<br>(11.0h-<br>30.5d)	4.2 days<br>(4.0h-<br>40.5d)	3.8 days<br>(3.0h-<br>37.4d)	3.0 days<br>(3.0h-<br>41.0d)	2.8 days<br>(3.0h-<br>99.3d)	2.9 days<br>(2.0h-<br>28.4d)	3.6 days<br>(8.0h-<br>107.5d)	3.2 days<br>(2.0h-<br>107.5d)
**Any respiratory support during 1st hour of ICU admission‡ §**<br>*Number (percent)*	2 (18.2)	8 (42.1)	12 (66.7)	4 (25.0)	6 (50.0)	7 (30.4)	27 (67.5)	6 (54.6)	19 (54.3)	16 (61.5)	34 (61.8)	30 (60.0)	41 (50.0)	20 (66.7)	37 (50.0)	48 (51.1)	38 (45.2)	355 (52.2)^
**Any respiratory support during ICU admission‡ §**<br>*Number (percent)*	5 (45.5)	10 (52.6)	10 (66.7)	9 (60.0)	8 (72.7)	11 (64.7)	29 (72.5)	5 (50.0)	22 (75.9)	16 (69.6)	30 (71.4)	31 (68.9)	48 (71.6)	22 (73.3)	53 (71.6)	57 (60.6)	52 (61.9)	418 (66.8)^
**Invasive respiratory support during ICU admission****<br>*Number (percent)*	5 (45.5)	9 (47.4)	13 (72.2)	7 (43.8)	6 (50.0)	13 (56.5)	39 (60.9)	5 (45.5)	21 (60.0)	11 (42.3)	37 (67.3)	30 (60.0)	39 (47.6)	16 (53.3)	39 (52.7)	40 (42.6)	31 (36.9)	361 (51.3)
**Length of invasive respiratory support***<br>Median (range)	4.7 days<br>(25.8h -<br>11.7d)	2.8 days<br>(1.3h -<br>11.2d)	2.7 days<br>(0.7h -<br>19.0d)	6.4 days<br>(12.2h -<br>11.7d)	2.1 days<br>(6.0h -<br>13.0d)	4.8 days<br>(4.5h -<br>27.2d)	5.4 days<br>(3.6h -<br>20.5d)	3.8 days<br>(29.3h -<br>22.5d)	7.0 days<br>(13.3h -<br>43.0d)	3.0 days<br>(1.8h -<br>9.8d)	2.6 days<br>(2.7h -<br>30.4d)	5.3 days<br>(15.2h -<br>31.3d)	4.9 days<br>(1.3h –<br>34.4d)	6.5 days<br>(1.9h –<br>41.1d)	3.8 days<br>(4.0h -<br>25.8d)	1.8 days<br>(0.2h –<br>27.5d)	4.8 days<br>(1.9h -<br>107.5d)	4.3 days<br>(0.2h -<br>107.5d)
**Deaths**<br>*Number (percent of cases) with co-morbidities*<br>*Number (percent of cases) without co-morbidities*	0 (0)<br>0 (0)	0 (0)<br>0 (0)	0 (0)<br>1 (6.7)	0 (0)<br>1 (10.0)	0 (0)<br>0 (0)	0 (0)<br>1 (5.3)	3 (9.7)<br>4 (12.1)	0 (0)<br>0 (0)	1 (7.1)<br>1 (4.8)	0 (0)<br>1 (7.7)	1 (7.1)<br>2 (4.9)	0 (0)<br>1 (3.1)	2 (5.3)<br>0 (0)	1 (9.1)<br>1 (5.3)	2 (5.0)<br>0 (0)	2 (4.8)<br>1 (1.9)	1 (3.3)<br>0 (0)	13 (4.5)<br>14 (3.4)
**Predominant influenza type (subtype) ††**	A(H3N2)	A(H3N2)	A(H3N2)	A(H3N2)	A(H1N1)	A(H3N2)	A(H3N2)	A(H3N2)	A(H3N2)	A(H3N2)	A(H3N2)	B‡‡	A(H1N1)	A(H1N1)	A(H1N1)	A(H3N2)	A(H1N1)	—

* h=hours; d=days; m=months; y=years

† Hospitalisation duration data missing for 98 cases

‡ Respiratory support is defined as any intervention to support respiratory function and includes both non-invasive and invasive methods. Some patients may have required combinations of both invasive and non-invasive support during their ICU admission. Non-invasive respiratory support includes: continuous positive airway pressure, biphasic positive airway pressure, negative pressure ventilation, high flow nasal cannula.

§ Respiratory support in 1st hour data missing for 24 cases; Respiratory support during admission data missing for 78 cases.

** Invasive respiratory support is mechanical ventilation delivered by endotracheal intubation or tracheostomy.

†† Compiled from annual and fortnightly reports from the Australian National Influenza Surveillance Scheme (available from www.health.gov.au/flureport). The predominant type/subtype listed had the highest cumulative number of reported cases nationally at the end of each season, based on diagnostic tests from all healthcare settings. As such, the predominant influenza type/subtype among paediatric ICU admissions may have differed.

‡‡ During this season, influenza B Victoria and Yamagata lineages were present in approximately equal proportions (See: Kaczmarek M, Owen R, Barr IG. Annual report of the National Influenza Surveillance Scheme, 2008. Communicable Diseases Intelligence. 2010;34(1):8-22.
